# Establishment of HLA class I and MICA/B null HEK-293T panel expressing single MICA alleles to detect anti-MICA antibodies

**DOI:** 10.1038/s41598-021-95058-8

**Published:** 2021-08-03

**Authors:** Ji-Ho Jeon, In-Cheol Baek, Cheol-Hwa Hong, Ki Hyun Park, Hyeyoung Lee, Eun-Jee Oh, Tai-Gyu Kim

**Affiliations:** 1 Department of Microbiology, College of Medicine, The Catholic University of Korea, 505, Banpo-Dong, Seocho-Gu, Seoul, 137-040 Korea; 2 Hematopoietic Stem Cell Bank, College of Medicine, The Catholic University of Korea, 505, Banpo-Dong, Seocho-Gu, Seoul, 137-040 Korea; 3 Departments of Laboratory Medicine, Seoul St. Mary’s Hospital, College of Medicine, The Catholic University of Korea, 505, Banpo-Dong, Seocho-Gu, Seoul, 137-040 Korea; 4 Department of Laboratory Medicine, International St. Mary’s Hospital, College of Medicine, Catholic Kwandong University, 25, Simgok-ro 100beon-gil, Seo-gu, Incheon, 22711 Korea

**Keywords:** Biological techniques, Immunology, Engineering

## Abstract

Pre- and post-transplantation anti-MICA antibody detection development are associated with an increased rejection risk and low graft survival. We previously generated HLA class I null HEK-293T using CRISPR/Cas9, while MICA and MICB genes were removed in this study. A panel of 11 cell lines expressing single MICA alleles was established. Anti-MICA antibody in the sera of kidney transplant patients was determined using flow cytometric method (FCM) and the Luminex method. In the 44 positive sera, the maximum FCM value was 2879 MFI compared to 28,135 MFI of Luminex method. Eleven sera (25%) were determined as positive by FCM and 32 sera (72%) were positive by the Luminex method. The sum of total MICA antigens, MICA*002, *004, *009, *019, and *027 correlation showed a statistically significant between the two methods (*P* = 0.0412, *P* = 0.0476, *P* = 0.0019, *P* = 0.0098, *P* = 0.0467, and *P* = 0.0049). These results demonstrated that HEK-293T-based engineered cell lines expressing single MICA alleles were suitable for measuring specific antibodies against MICA antigens in the sera of transplant patients. Studies of antibodies to MICA antigens may help to understand responses in vivo and increase clinical relevance at the cellular level such as complement-dependent cytotoxicity.

## Introduction

The major histocompatibility complex (MHC) region, which plays an important role in protecting against pathogens, includes both polymorphic and multi-copy genes^1^. The MHC class I chain-related gene A (MICA), which is located on human chromosome 6, approximately 46.4 Kb centromeric to the HLA-B locus, encodes a protein with three extracellular domains, a transmembrane (TM) segment, and a carboxy-terminal cytoplasmic tail^2,3^. MICA polymorphisms have been identified in over 220 alleles in the IPD-IMGT/HLA database release 3.43.0 (http://www.ebi.ac.uk/imgt/hla). MICA is found in many cell lines as well as in primary cells such as endothelial cells, fibroblasts, and gastrointestinal epithelial cells, and cell stress can induce its expression^4^. MIC proteins are stress markers for cells, because the expression of these molecules is induced by heat, viral infection, inflammation, and DNA damage^5–9^. The extracellular domains consist of α1, α2, and α3 chains that flexibly function as ligands for natural killer (NK) group 2D (NKG2D) receptor expressed in NK cells, γδ T cells, and αβ CD8 T cells^10^. It is also expressed in several epithelial tumors, including lung, breast, kidney, ovarian, prostate, and colon carcinomas^11^.

The pretransplant crossmatch, which involves testing the recipient’s serum for cytotoxicity against the donor cells (lymphocytes), was introduced in the early days of renal transplantation^12^. Antibody-mediated allograft injury caused by donor HLA-specific antibodies (DSA) has been identified as one of the major causes of late graft loss^13,14^. Several techniques are currently used to detect anti-HLA antibodies to decrease the rate of organ rejection and improve survival, including cell-based methods such as complement-dependent cytotoxicity (CDC), flow cytometric method (FCM), and solid-phase-based methods such as enzyme-linked immunosorbent assay (ELISA), and Luminex.

The MICA gene is highly polymorphic, and exposure to mismatched MICA antigens can induce allogenic responses that are associated with transplant failure^15–17^. Pretransplant anti-MICA antibodies have been demonstrated to be risk factors for allograft rejection and lower graft survival^17–23^. Also, the post-transplantation development of anti-MICA antibodies is associated with acute rejection and allograft loss^21,22,24^. In an earlier study, a single MICA gene was expressed and used in a CR1 cell line that does not express HLA, but it was found that the CR1 cell line also expressed its own endogenous MICA gene^25^. An insect-derived cell line, High Five cells, was tried to be used, but it was judged unsuitable because of the nonspecific heterologous antibody response to carbohydrate molecules^26^. Since there is no human cell line in which the MICA/B gene is not fully expressed, the study of anti-MICA antibody assay for single MICA antigen using cell lines could not be continued. Currently, the Luminex method using ELISA or beads using soluble recombinant protein has been mainly developed and used clinically. Recently, because antibody measurement using Luminex assay is too sensitive, the clinical relevance is being questioned^27,28^. To determine the titer of a clinically meaningful antibody, it must be compared with the antibody response to MICA expressed on the cell surface. Therefore, in the previous study, a single MICA gene was transduced into HEK-293T cell line from which HLA class I had been completely removed and used in this study after MICA was completely removed.

We previously generated the HEK-293T cell line from which the HLA class I gene was removed using CRISPR/Cas9^29^. In this study, we generated the cell line from which MICA and MICB genes were removed. Based on this cell line, a panel of cell lines that expresses 11 types of MICA alleles frequent in Koreans was established. The presence of anti-MICA alloantibodies in the sera of kidney transplant patients was determined by flow cytometry method (FCM) using this panel of cell lines expressing single MICA alleles and FCM was also compared with Luminex method for single antigen.

## Results

### Establishment of MICA cell lines expressing single MICA antigen

**Figure 1:**
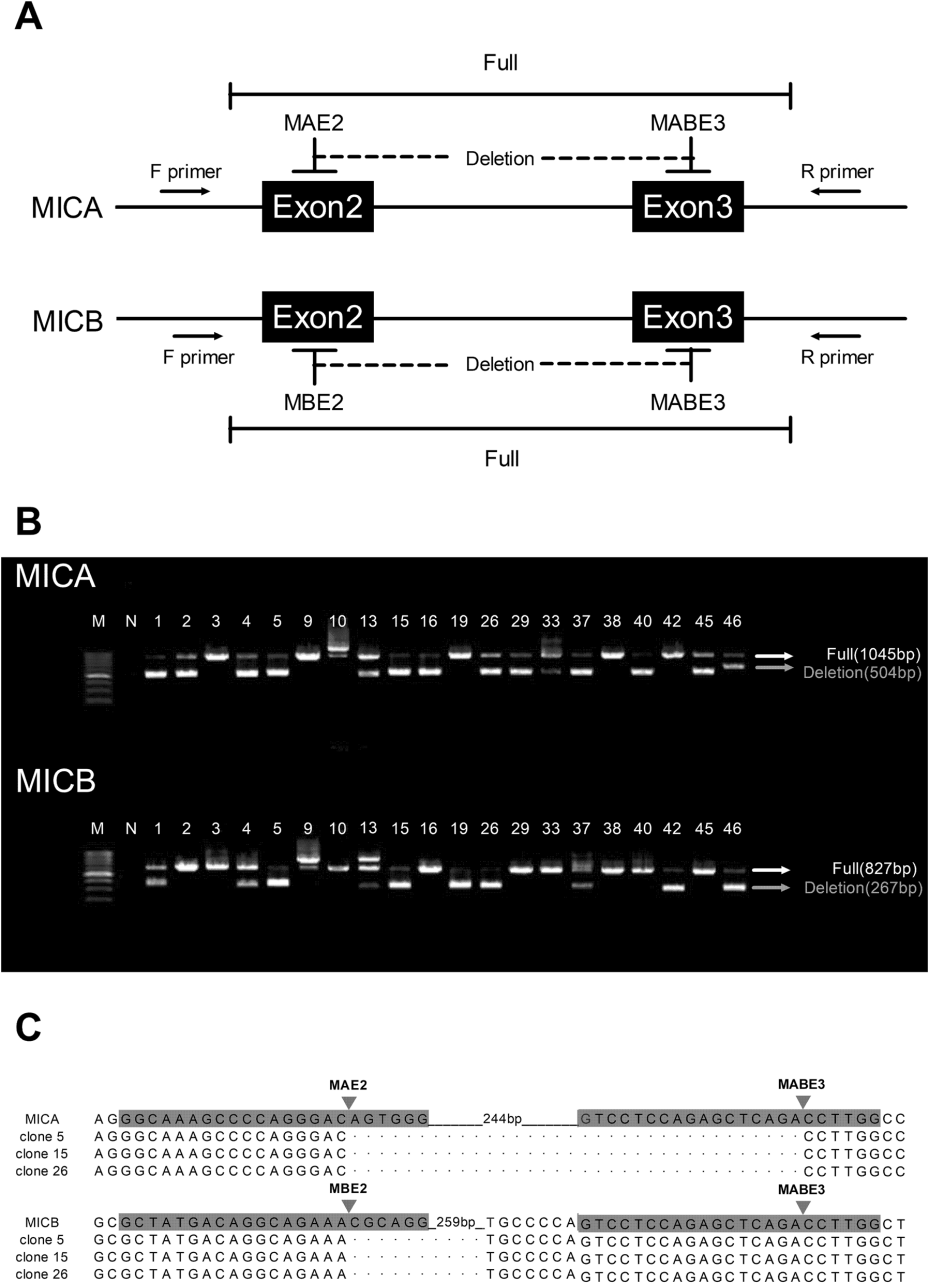
Additional deletion of both MICA and MICB genes on HLA class I deleted HEK-293T cell line (H1E-25) using the multiplex CRISPR/Cas9 system. (**A**) Three guide RNA (gRNA)-Cas9 plasmids (MAE2, MBE2, and MABE3) were designed for deletions between exons 2 and 3 of each MICA and MICB gene. (**B**) For detection of deletion mutation, targeted PCR were performed using specific primer pairs that included exons 2 and 3 of MICA/B genes, and genotypes were analyzed using gel electrophoresis. The black arrow indicates the PCR product size of the wild-type 293T control sample. The gray arrow indicates the PCR product size of the predicted deletion between exons 2 and 3 by designed gRNAs. When the PCR product size was different from that in control cells, we designated the detectable changes in PCR products as deletions (shorter products), insertions (longer products), or lack of amplification. When PCR products were detected as single or double bands, they were regarded as homozygous or heterozygous. (**C**) Nucleotide sequences between exons 2 and 3 in the selected MICA/B null clones (clones 5, 15, and 26). The target guide RNA sequence (in gray color), cut site (inverted triangle), and removed sequence (dot) were aligned to the nucleotide sequence from wild-type HEK-293T cell line.

We selected the H1E-25 HEK-293T cell line from which HLA class I was deleted to rule out alloantibody reactions against HLA^29^. Three plasmids encoding the Cas9 protein and gRNA to target MICA exon 2 (MAE2), MICB exon 2 (MBE2), and MICA/B exon 3 (MABE3) were used for complete deletion of the MICA/B molecule (Fig. 1A). These three plasmids were co-transfected with H1E-25 HEK-293T cells. Cells that were successfully transfected and expressed fluorescence were separated into single cells using fluorescence-assisted cell sorting (FACS) and cultured into 96-well plates. For the 48 clones growing, the cell lines with predicted deletions were investigated via PCR of the regions containing exon 2 and exon 3 (Fig. 1B). There were 12 heterozygous and six homozygous detectable changes (DCPs) in MICA and 14 heterozygous and 13 homozygous DCPs in MICB. We found that exon 2–3 of MICA/B was completely deleted in clones H1ME-5, -15, and -26 (Fig. 1C). MICA was cut at the predicted cut sites, and 267 bp were deleted from all three clones. However, in the exon 3 site of MICB, a total of 265 bp was deleted from all three clones by cutting 24 bp ahead of the predicted cut site.

**Figure 2:**
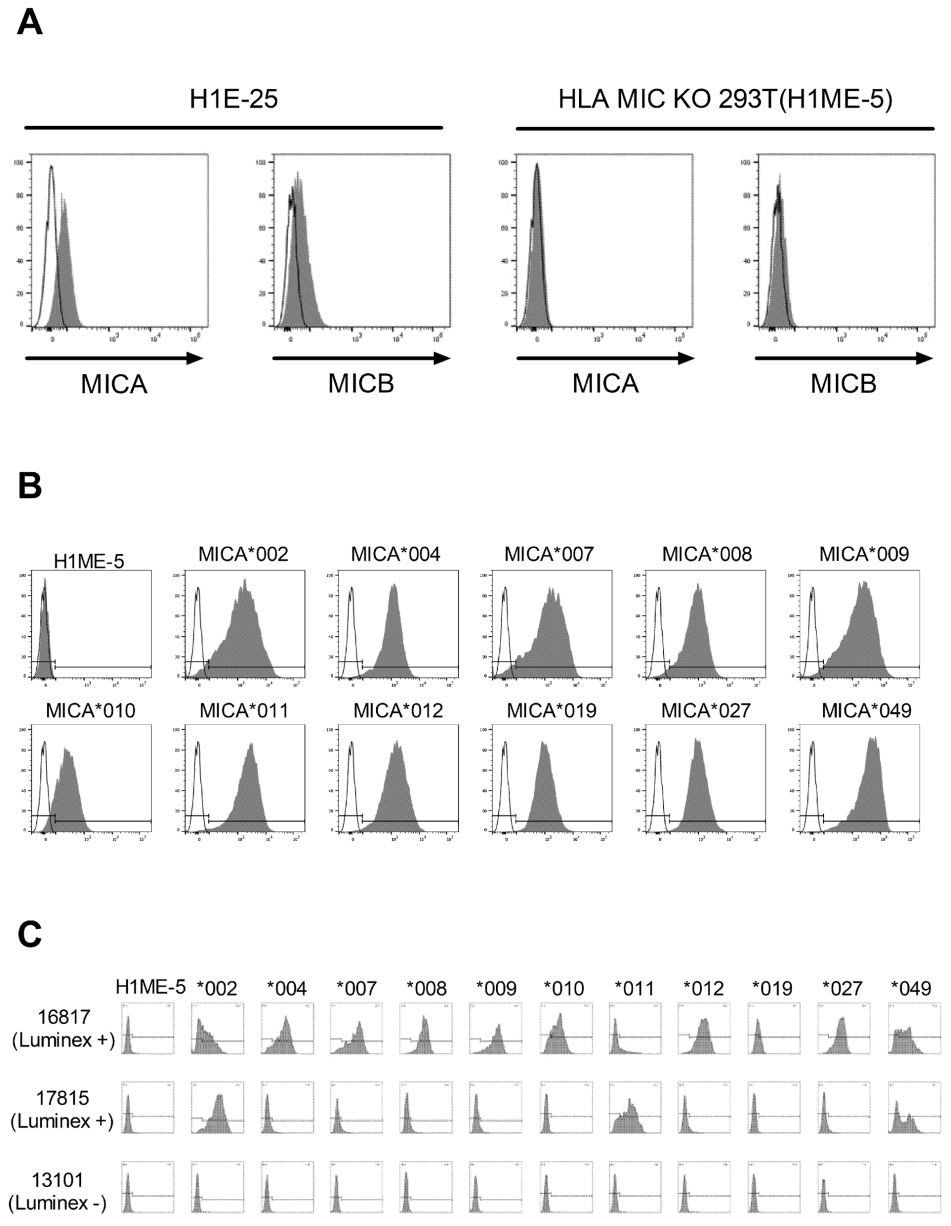
Establishment of cell lines expressing a single MICA allele using HEK-293T cell line (H1ME-5) from which both MICA and MICB genes have been deleted in addition to the HLA class I genes (H1E-25). (**A**) The expression of surface MICA and MICB molecules in H1E-25 and H1ME-5 HEK-293T cell lines. (**B**) Establishment of cell lines expressing single MICA allele from H1ME-5 HEK-293T cell line. Transduction and cloning using lentivirus vectors expressing each MICA allele genes, including MICA*002, *004, *007, *008, *009, *010, *011, *012, *019, *027, and *049. The MICA molecules expressed on the surface of selected cells were confirmed by anti-MICA PE (gray) antibody. (**C**) Representative response patterns of single MICA allele-expressing cell lines to the sera of two positive (16,817 and 17,815 MFI) and one negative (13,101 MFI) serum confirmed by the Luminex method.

Although MICA and MICB were weakly expressed in the H1E-25 HEK-293T cell line, it was confirmed that they were not expressed at all in the three clones from which both MICA and MICB were removed. In the subsequent experiments, the selected H1ME-5 HEK-293T cell line was used (Fig. 2A). To establish a cell line panel expressing single MICA alleles, H1ME-5 HEK-293T cells were transduced with lentiviruses encoding 11 MICA alleles, MICA*002, *004, *007, *. 008, *009, *010, *011, *012, *019, *027, and *049. MICA-positive cells were sorted and cultured on day 6 after transduction. FACS analysis confirmed the high expression of 11 single MICA alleles (Fig. 1B). To investigate whether these cell panels were suitable for measuring anti-MICA antibodies in the patient serum, the serum determined as positive and the serum determined as negative were subjected to flow cytometric method (FCM) after fluorescence staining (Fig. 2C). Negative serum (13,101 MFI) was not measured in all cells expressing a single MICA allele in the same manner as H1ME-5, which did not express MICA. Positive serum (17,815 MFI) responded only to certain MICA alleles such as MICA*002, *011, and *049. These results indicate that the non-specific response of serum in the cell panel based on H1ME-5 HEK-293T cells is very low.

### Responses of FCM and Luminex method to MICA alleles in patient serum

In this study, 11 MICA antigens used for FCM were selected to be present at a frequency of 2% or more in Koreans (Table 1). A total of 15 MICA antigens were included in the Luminex method, but six antigens were not reported in Koreans, and two antigens had a frequency of less than 1%. Seven antigens were detected by both the FCM and Luminex methods. Because MICA*008 and *027 or *009 and *049 alleles showed the same amino acid sequences of the extracellular domain on the cell surface, the Luminex method includes only beads of MICA*027 and of MICA*009. However, FCM includes cell lines expressing all these single antigens.

**Table 1:** MICA allele frequencies in Koreans and MICA alleles included in cell lines expressing single MICA allele for FCM and Luminex method.

MICA alleles	Frequencies 2N = 400, N (%)	FCM	Luminex
MICA*001	0		v
MICA*002:01	42 (10.5)	v	v
MICA*004	30 (7.5)	v	v
MICA*005	0		v
MICA*006	3 (0.8)		v
MICA*007:01/02	13 (3.3)	v	v
MICA*008:01/02^a^	85 (21.3)	v	
MICA*009:01	17 (4.3)	v	v
MICA*010:01	69 (17.3)	v	
MICA*011	9 (2.3)	v	
MICA*012:01	40 (10)	v	v
MICA*015	0		v
MICA*018	0		v
MICA*017	2 (0.5)		v
MICA*019	11 (2.8)	v	v
MICA*027	43 (10.8)	v	v
MICA*028	0		v
MICA*045	7 (1.8)		
MICA*046	0		v
MICA*049^b^	29 (7.3)	v	

*Korean frequencies of MICA alleles have been reported in previous study^33^.

*Each pairs of the marks, ^a^(MICA*008:01 and *027) and ^b^(MICA*009:01 and *049) have the same amino acid sequences of extracellular domain.

**Figure 3:**
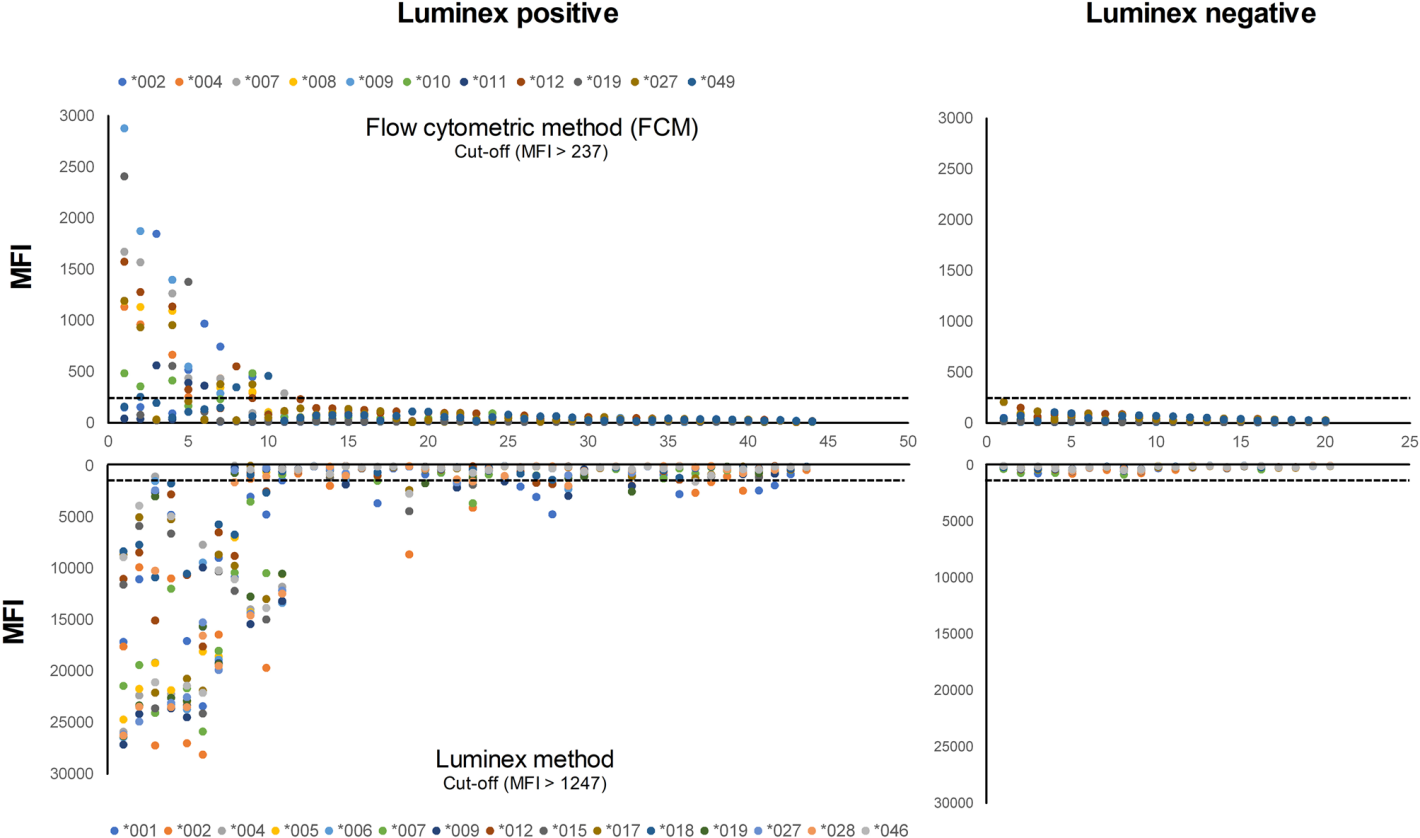
Comparison of response patterns to each MICA allele by flow cytometric method (FCM) and Luminex method using sera of kidney transplant patients (n = 64). Results were displayed in the order of sera with weak response to strong response. As for the Luminex method, 33 sera with a response of 1247 MFI or higher were determined as positive, and, as for the FCM, 11 sera with a response of 237 MFI or higher were determined as positive.

The 64 sera collected from kidney transplant patients used in this study consisted of 44 positive sera and 20 negative sera using the Luminex screening kit (mixed) (Fig. 3). The cut-off value for the FCM test was calculated as mean + 4 SD based on the data on the allele antigen with the largest deviation from 20 negative sera and determined as 237 MFI (mean = 59, SD = 45). Similarly, the cut-off value for the Luminex method was determined to be 1247 MFI (mean = 283, SD = 241). Among the negative sera, the maximum values for FCM and Luminex methods were 209 and 822 MFI, respectively.

After testing the positive 44 sera using the FCM and Luminex methods, the results from FCM were sorted from the highest value based on the allele with the highest response in each serum, and the results from the Luminex method were listed corresponding to FCM (Fig. 3). The maximum value of FCM was 2879 MFI, which was approximately 1/10 of the 28,135 MFI of the Luminex method. Eleven sera with more than this cut-off value were determined to be positive by FCM. Based on the above cut-off value, there were 11 (25%) and 32 (72%) positive serum samples by FCM and Luminex method, respectively.

### Distribution of positive responses according to individual MICA antigens

**Figure 4:**
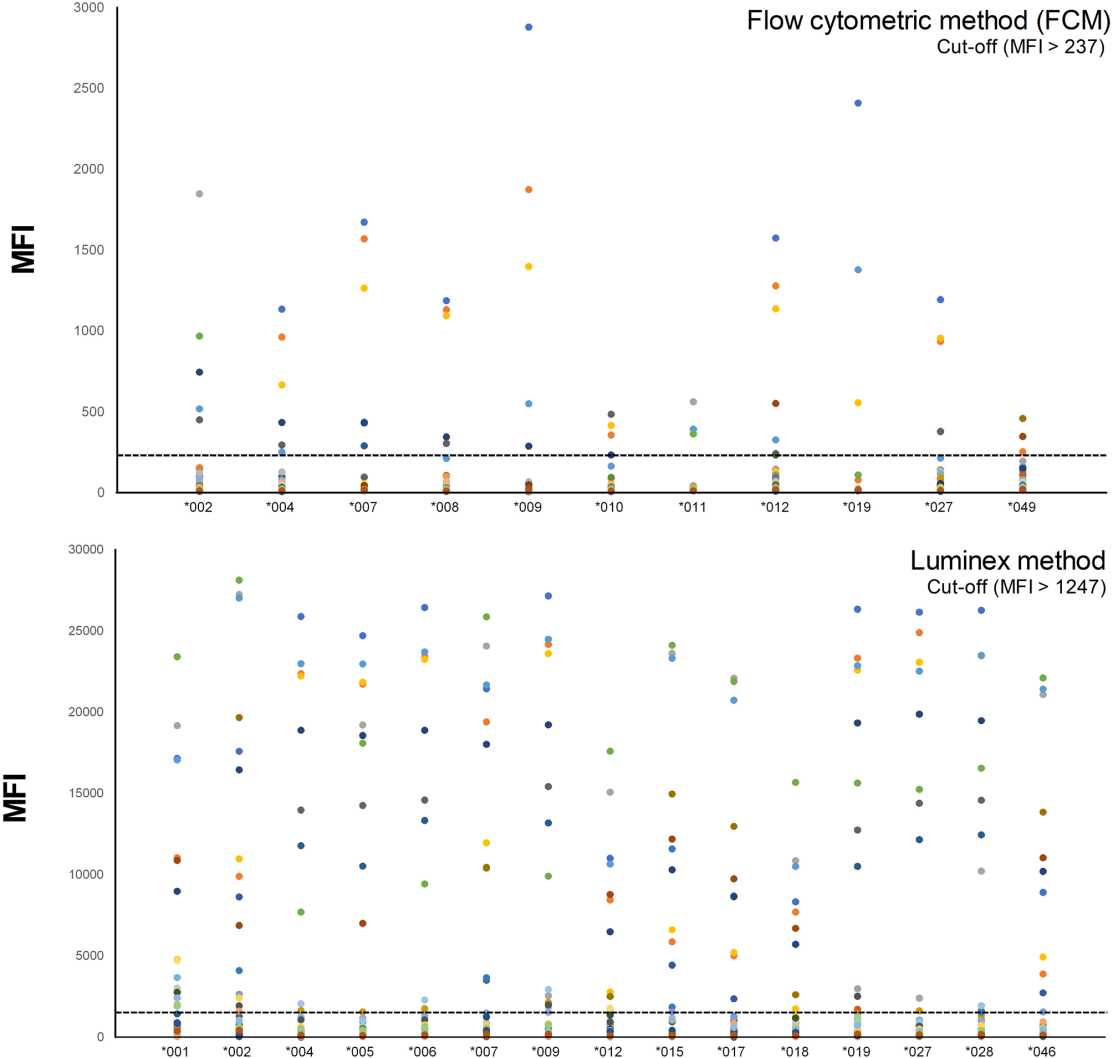
Response analysis for each MICA allele and flow cytometric method (FCM), Luminex method using positive sera from kidney transplant patients (n = 44). Both FCM and Luminex method displayed serum responses in allele order. As for Luminex method, the allele showing the strongest response was MICA*002, and, as for FCM, MICA*009 was identified as the allele showing the strongest response.

In the analysis of responses on individual MICA antigens (Fig. 4), the order of MICA antigens that showed strong responses (mean) in the FCM test was *009 (179 MFI), *012 (169 MFI), *007 (147 MFI), *002 (143 MFI), *027 (133 MFI), *008 (124 MFI), *004 (115 MFI of), *019 (115 MFI), *049 (79 MFI), *010 (78 MFI), and *011 (45 MFI). The order of MICA antigens in the Luminex method was *002 (4477 MFI), *005 (4333 MFI), *028 (4209 MFI), *007 (4190 MFI), *009 (4044 MFI), *019 (3929 MFI), *027 (3920 MFI), *006 (3849 MFI), *004 (3623 MFI), *001 (3427 MFI), *015 (3409 MFI), *046 (2957 MFI), *017 (2925 MFI), *012 (2199 MFI), and *018 (1858 MFI).

### Correlation between the results of FCM and Luminex method

**Figure 5:**
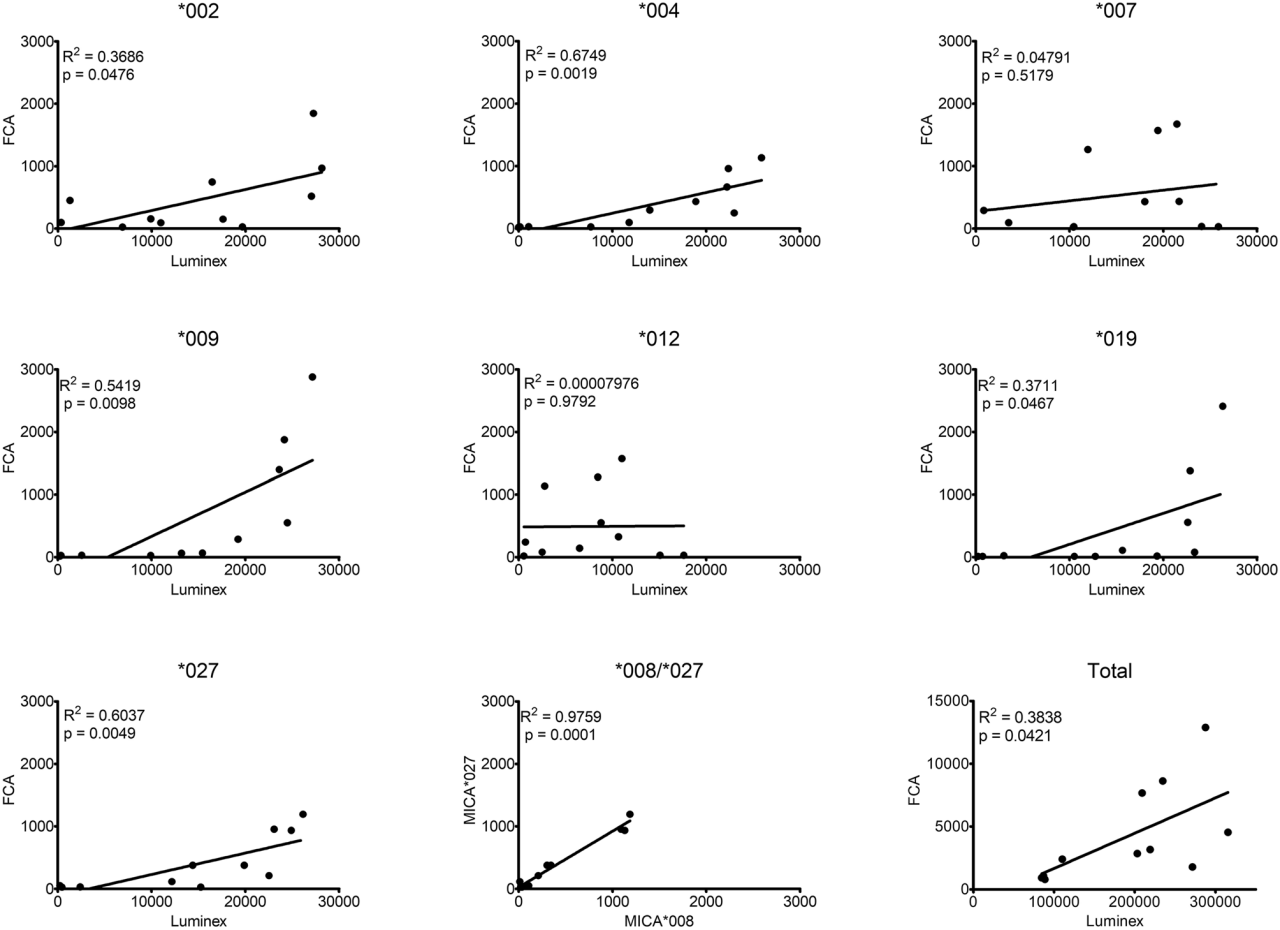
Correlation of total and each MICA allele response for FCM and Luminex method using 11 positive sera. The total is represented by the sum of the responses of the included alleles for each assay. Correlation was analyzed only for MICA alleles in common for both methods.

The correlation between the results of the two assays was analyzed in 11 serum samples that were positive in the FCM assay (Fig. 5). First, the total response in each serum sample was compared for each of the seven individual antigens present in both assays. MICA*002, *004, *009, *019, and *027 correlation showed a statistically significant (*P* = 0.0412, *P* = 0.0476, *P* = 0.0019, *P* = 0.0098, *P* = 0.0467, and *P* = 0.0049, respectively). However, the MICA*007 and *012 alleles showed no significant correlation.

### Specificities to single MICA alleles of 11 positive sera by FCM

When analyzing the response pattern to each antigen for 11 positive sera in the FCM test, six sera were positive for more than five antigens, three were positive for two antigens, and three were positive for a single antigen (Table. 2). Two sera positive for a single antigen showed specific reactions to MICA*007 and *049, respectively. Two of the three sera positive for the two antigens reacted equally to MICA*002 and *011. In the sera positive for eight antigens, two sera responded identically to MICA*004, *007, *008, *009, *010, *012, and *027. MICA*008 and *027, which had the same ECD amino acid sequence, showed the same reaction (Fig. 5). However, MICA*009 and *049, which have the same ECD amino acid sequence, did not show the same reaction. The reactions to MICA*010 and *011, which existed in Koreans but were not included in the Luminex method, were 36% and 27%, respectively.

**Table 2:** Specificities to single MICA alleles of 11 positive sera.

Serums	Positive alleles	FCM MICA alleles (N = 11, MFI)										
(N = 11)	N (%)	*002	*004	*007	*008	*009	*010	*011	*012	*019	*027	*049
**17498**												
FCM	8 (73)	149	*1134*	*1672*	*1187*	*2879*	*484*	41	*1574*	*2409*	*1193*	157
Luminex	7 (100%)	*17,603*	*25,893*	*21,446*	NA	*27,164*	NA	NA	*11,014*	*26,340*	*26,158*	NA
**17322**												
FCM	8 (73)	92	*666*	*1264*	*1094*	*1398*	*414*	35	*1137*	*556*	*955*	53
Luminex	7 (100%)	*10,982*	*22,227*	*11,966*	NA	*23,622*	NA	NA	*2783*	*22,602*	*23,078*	NA
**16817**												
FCM	8 (73)	154	*962*	*1570*	1131	*1874*	*356*	38	*1279*	78	*934*	*253*
Luminex	7 (100%)	*9901*	*22,370*	*19,412*	NA	*24,173*	NA	NA	*8446*	*23,335*	*24,906*	NA
**17649**												
FCM	7 (64)	*518*	*251*	*436*	210	*549*	164	*392*	*326*	*1378*	212	107
Luminex	7 (100%)	*27,030*	*22,994*	*21,681*	NA	*24,496*	NA	NA	*10,653*	*22,869*	*22,530*	NA
**12862**												
FCM	6 (55)	*745*	*433*	*432*	344	*287*	232	11	142	19	*378*	148
Luminex	7 (100%)	*16,457*	*18,894*	*18,025*	NA	*19,227*	NA	NA	*6493*	*19,337*	*19,886*	NA
**12816**												
FCM	5 (45)	*450*	*295*	96	304	66	*484*	9	241	13	*377*	65
Luminex	6 (86)	*1285*	*13,979*	*3504*	NA	*15,427*	NA	NA	717	*12,753*	*14,397*	NA
**17185**												
FCM	2 (18)	*1847*	30	35	35	32	27	*562*	32	24	32	195
Luminex	6 (86)	*27,249*	1069	*24,077*	NA	*2554*	NA	NA	*15,081*	*2987*	*2407*	NA
**17609**												
FCM	2 (18)	*968*	27	30	35	29	35	*363*	30	109	27	133
Luminex	7 (100%)	*28,135*	*7702*	*25,877*	NA	*9914*	NA	NA	*17,610*	*15,647*	*15,257*	NA
**12547**												
FCM	2 (18)	24	22	24	20	17	20	13	*551*	13	26	*347*
Luminex	3 (43%)	6880	0	10,412	NA	245	NA	NA	8784	717	448	NA
**12610**												
FCM	1 (9%)	26	28	32	106	28	86	11	80	13	50	*458*
Luminex	0 (0%)	1017	312	710	NA	379	NA	NA	559	1072	609	NA
**12986**												
FCM	1 (9%)	97	99	289	13	62	53	9	20	13	115	22
Luminex	4 (57%)	347	11,788	866	NA	13,185	NA	NA	552	10,514	*12,163*	NA
**Positive sera**												
FCM	N (%)	5 (45)	6 (55)	6 (55)	5 (45)	5 (45)	4 (36)	3 (27)	5 (45)	3 (27)	5 (45)	3 (27)
Luminex	N (%)	9 (82)	8 (73)	9 (82)	NA	9 (82)	NA	NA	8 (73)	9 (82)	9 (82)	NA

*Values marked in italics indicate a positive reaction as more than the cut-off value.

*NA* not applicable.

## Discussion

In the initial study on allogeneic antibodies against MICA antigens, a cell line expressing a single MICA antigen was generated using m-HMy2. The C1R cell line weakly expresses the HLA-C gene but does not express HLA-A, HLA-B, or MICA. It was used for the detection of antibodies against MICA antigen reaction through CDC assay or for identification of shared epitopes by antibody absorption in patient sera^24,30^. The K562 cell line, which does not express HLA, expresses a single HLA antigen and is used for FCM or CDC tests. However, MICA was expressed and was not used for studies on a single MICA antigen^31^. High Five insect cells that do not express human alloantigens were also used to generate single MICA-expressing cell lines, but the recombinant glycoproteins on the cell surface were found to cause cross-reactions with antibodies in human serum^26^. As it was not possible to obtain a cell line expressing a single MICA antigen, recombinant soluble proteins with extracellular domains of each MICA antigen produced in *Escherichia coli* were mainly used for ELISA or Luminex method from the beginning^15,32^. We previously used the CRISPR/Cas9 to remove all the HLA-A, -B, and -C genes of the HEK-293T cell line to establish a cell line without allogeneic response to HLA^29^. In this study, MICA and MICB genes were removed to establish a cell line without both HLA and MICA/B genes (Fig. 2). This cell line confirmed that non-specific reactions did not occur in either the positive or negative sera of all patients and was used as internal negative control in this study.

As DNA typing methods for MICA have been developed precisely, more MICA alleles have been reported worldwide, and the distribution of the MICA allele in Koreans has also been well investigated^33^. In this study, 11 types of MICA alleles with a frequency of 2% or more in Koreans were selected to generate cell lines expressing a single MICA antigen for FCM. Compared to the currently clinically used Luminex method, antibody responses to MICA*010 and *011 antigens, which were not included in the Luminex method, were found to be 36% and 27%, respectively (Table 2). MICA*001, *005, *015, *018, *028, and *046 antigens included in the Luminex method were not present in Koreans, but reactive antibodies were present (Fig. 4). This phenomenon is known to be due to cross-reactions between MICA antigens that share common epitopes^30^. For example, an antibody against MICA*001 cross-reacts with MICA*001, *012: 01, *018: 01, *007: 01, *002: 01, and *017 alleles belonging to antigen group 1, which shares the same epitope. In FCM, six out of 11 antibodies in the positive sera showed strong cross-reactivity against five or more antigens. There was no significant correlation between *007 and *012 alleles in the serum correlation study for the antigens detected by FCM and Luminex method. Comparing the results of FCM and Luminex is thought to be influenced by various factors. For FCM, Anti-human IgG Fc-FITC was used, and for LABscreen MICA single antigen, PE–Conjugated Goat Anti-Human IgG was used to confirm the MFI of each serum. Although the amount of antigen used in FCM and Luminex method was not accurately compared, it is thought that there is a relationship between the target amount and the measurement of antibody. Also, it is thought that the sensitivity of the measuring equipment used for FCM and Luminex will also have an effect (Fig. 5). Since MICA*008 and *027 alleles had the same extracellular domain, *008 was not included in the Luminex method, but antibody responses to both antigens were measured by FCM as expected (Table 2). However, MICA*009 and *049 alleles differ in one amino acid sequence in the intracytoplasmic region in exon 6, and the antibody responses are inconsistent, even though the extracellular domains are identical. These results may be due to the fact that MICA antigens are expressed on the cell surface in FCM, but, in the Luminex method, they are recombinant soluble proteins attached to beads. Previous studies on anti-HLA antibody suggest that conformational changes can occur due to the attachment of recombinant soluble HLA molecules to the beads^31^. In addition, when antibodies are measured using cell lines, non-specific responses to cell surface molecules other than MICA cannot be excluded. More sophisticated studies are needed in the future on these issues.

Initially, in the field of organ transplantation, tests to measure antibodies for histocompatibility mainly used the CDC assay and FCM, but the sensitivity increased dramatically as solid phase assays such as ELISA or Luminex method were developed. These sensitive tests can increase the early detection of patients at risk for transplant rejection, but low levels of antibodies may not be relevant in clinical events. In this study, the Luminex test was more sensitive than the FCM test, and only few serum positives by the Luminex method were found to be positive by FCM (Fig. 3). All 11 serum samples positive by FCM showed more than 10,000 MFI by the Luminex method. Pre-transplant donor-specific HLA antibodies measured by both the CDC and Luminex methods in Korean kidney transplant patients were identified as risk factors for microvascular inflammation in allograft biopsy, but anti-MICA antibodies measured using the Luminex method did not reach statistical significance^34^. In a study on prevalence of MICA antibodies in renal biopsies after transplantation, the MFI value was significantly higher in patients with interstitial fibrosis and tubular atrophy (IFTA) II or III (n = 3, median ± SE, 21,919.0 ± 2581.0) than in patients without IFTA II or III (n = 9, median ± SE, 500.0 ± 155.8) (*P* = 0.009)^35^.

The results of this study confirmed that the HEK-293T cell line, in which both HLA and MICA/B genes were removed using CRISPR/Cas9 technology, was suitable for measuring antibodies against single MICA antigens in the serum of transplant patients. In FCM, antibodies were tested only for IgG, but simultaneous measurement of IgM and other immunoglobulins and other subclass antibodies may be required. Studies on antibodies against MICA antigens at the cellular level such as CDC and antibody-dependent cytotoxicity in NK cells may help to better understand responses in vivo and increase clinical relevance. Following this study, studies to measure antibodies against MICB antigens are being conducted, and it is expected that the same approach may be applied on various non-HLA antigens in the future.

## Methods

### Cell culture

MICA alleles of peripheral blood mononuclear cells (PBMCs) were selected using SBT at the Catholic Hematopoietic Stem Cell Bank. All cell lines HEK-293T (CRL-3216; ATCC, Manassas, VA, USA), HLA class I null-293T (H1E-25), HLA class I, and MICA/B null-293T (H1ME-5) were cultured in Dulbecco’s modified Eagle’s medium (Lonza) supplemented with 10% fetal bovine serum (Hyclone, Logan, UT, USA), 1% l-glutamine (Lonza), and 1% penicillin–streptomycin (Lonza). Frozen cell lines were thawed, expression was confirmed by flow cytometry, and used in this study within 1 month. All cell lines were frozen in freezing media (DMSO:Media:FBS = 1:4:5).

### Sera of kidney transplant patients

Pre-tranplant sera were obtained from the kidney transplant recipients who were requested for PRA testing at Seoul St.Mary`s hospital from January 2014 to December 2016. A total of 64 kidney transplant samples were provided by the Department of Laboratory Medicine (The Catholic University of Korea, Seoul, Korea). Samples were marked with serial numbers and stored at − 20 °C. When used in the experiment, they were thawed at 25 °C and vortexed. All subjects provided informed consent to participate in the study. Also, written informed consent was obtained from each participant and their parents or guardians. This study protocol was approved by the Institutional Review Board (IRB) of the Catholic University of Korea (IRB Number: MC13SISI0126 [DNA], MC19SNSI0068 [serum]), Seoul, Korea, and conducted in accordance with the Declaration of Helsinki. First, the presence of MICA in the sera was analyzed using LABScreen Mixed (One Lambda Inc, Canoga Park, CA, USA).

### Deletion of both MICA and MICB genes using CRISPR/Cas9

Three guide RNAs (gRNAs) were used targeting MICA exon 2 (MAE2): 5′-GGCAAAGCCCCAGGGACAGTGGG-3′; MICB exon 2 (MBE2): 5′-GCTATGACAGGCAGAAACGCAGG-3′; MICA/B exon 3 (MABE3): 5′-GTCCTCCAGAGCTCAGACCTTGG-3′. All-in-one plasmids, including both gRNA and Cas9 genes, were obtained from Genscript (PX458 and PX459). H1E-25 cells were seeded at 2 × 10^6^ cells/10 mL in antibiotic-free Dulbecco’s modified Eagle’s medium (Lonza). Twenty-four hours later, a mixture of individual all-in-one plasmids specific for each of the targets was transferred to the H1E-25 cell line using Lipofectamine reagent (Invitrogen, Carlsbad, CA, USA). At 48 h after transfection, the cells were analyzed using flow cytometry. Six days after transfection, cells negative for MICA/B were sorted, and clones were established. To establish the MICA/B-null cell line, cells co-transfected with three plasmids were harvested in autoMACS Rinsing Solution (Miltenyi Biotec, Bergisch Gladbach, Germany) and stained with anti-MICA-PE (FAB1300P; R&D Systems, Minneapolis, MN, USA) and anti-MICB-APC (FAB1599A; R&D Systems) for 30 min at 4 °C. Live, GFP-negative, and MICA/B-negative 293T cells were sorted, and single cells were seeded in 96-well plates using MoFlo XDP cell sorters (Beckman). Positive cells were sorted, and single cells were seeded onto 96-well plates using MoFlo XDP cell sorter. At 2–3 weeks after sorting, single-cell clones were established.

### PCR and sequencing for detection of deleted MICA and MICB genes

Two–three weeks after sorting, MICA/B-negative single-cell clones were established and cultured on 6-well plates (n = 188). Clonal genomic DNA was isolated from 1 × 10^5^–10^6^ cells from each clone using a TIANamp Genomic DNA kit (Tiangen Biotech, Beijing, China) according to the manufacturer’s instructions. Amplification of each target region was carried out using PCR with forward and reverse primers: MICA, 5′-CGTTCTTGTCCCTTTGCCCGTGTGC-3′ (forward) and 5′-GAATTGGAGGGAGAGGAGAGC-3′ (reverse); MICB, 5′-AGCCCCACAGTCTTCGTTAC-3′ (forward) and 5′-CCAGGGTCGGTACCTGTTCT-3′ (reverse). The PCR program consisted of one cycle of 95 °C for 3 min, 35 cycles of 98 °C for 20 s, 65 °C for 15 s, and 72 °C for 20 s; one cycle of 72 °C for 2 min; and one cycle of 10 °C for 5 min. PCR products were analyzed by Gel Doc XR + system (Bio-Rad, Hercules, CA, USA) on 2% agarose gels, using SYBR Green and a 100-bp ladder (Bioneer, Daejeon, Korea) for screening of predicted large deletion clones. PCR products of selected clones were analyzed by Sanger sequencing (Cosmo Genetech, Seoul, Korea) using the same primers.

### Production of lentivirus expressing single MICA alleles

Commercially available kits were tested for PBMC RNA isolation using NucleoSpin RNA (Macherey–Nagel, GmbH, Duren, Germany), according to manufacturer protocols were followed for the kit. cDNA was synthesized using the SuperiorScript III cDNA Synthesis kit (Enzynomics, Daejeon, Korea). Amplification of each MICA allele was carried out using PCR with one forward and two reverse primers: 5′-ATGGGGCTGGGCCCGGTCTT-3′ (forward), 5′-CTAGGCGCCCTCAGTGGAGC-3′ (reverse 1), and 5′-CTAGGTGCCCTCAGTGGAGC-3′ (reverse 2). PCR was carried out in 30 μL reaction mixture in 200-μL PCR tubes (Axygen, Hangzhou, China), containing 50 ng genomic DNA, distilled water, 10 × PCR reaction buffer (Kapa Biosystem, MA, USA), 0.6 μL of each 2.5 mM dNTPs, 10 μM primer sets, and 1.5 U of Taq DNA polymerase (Kapa Biosystem). The restriction enzymes BspE1 (R108S; Enzynomics) and Sal1 (R009S; Enzynomics) were used to remove copGFP from the pCDH vector (#CD523A-1; SBI, Palo Alto, CA, USA). The EZ-Fusion Cloning kit (EZ015M; Enzynomics) was used for cloning. Plasmid sequencing was performed using Sanger sequencing (Cosmo Genetech). To produce lentiviruses encoding each molecule (MICA*002, *004, *007, *008, *009, *010, *011, *012, *019, *027, and *049), 5 × 10^6^ HEK-293T cells/10 were seeded in T75 flasks. Twenty-four hours later, 10 μg of a cloned MICA pCDH plasmid and lentivirus packaging plasmids (5 μg pMD2.G and 5 μg psPAX2, cat nos. #12259, #12260; Addgene, Cambridge, MA, USA) were co-transfected into HEK-293T cells using Lipofectamine reagent (Invitrogen). At 48 h after transfection, lentiviral supernatants were harvested and filtered through 0.45-μM filters.

### Generation of cell lines expressing single MICA alleles

For transduction of each lentivirus, 5 × 10^5^ 293T cells/mL were seeded into 6-well plates. Twenty-four hours later, 500 μL of lentiviral supernatant and 8 μg/mL of polybrene were added to 293T cell cultures. At 48 h after transduction, the cells were cultured and analyzed using flow cytometry. Anti-MICA-PE (FAB1300P; R&D Systems) was used. Target cells were harvested and stained with fluorescently labeled anti-human MICA antibodies for 30 min at 4 °C in the dark. Stained cells were analyzed using FACS Canto or Fortessa flow cytometer (BD Biosciences, San Jose, CA, USA).

### FCM for detection of anti-MICA antibodies in sera of patients

The recipient serum (5 μL) was incubated with 20 μL of the single MICA cell lines adjusted to 2 × 10^5^/mL at room temperature for 30 min. Anti-human IgG Fc-FITC (F031501; DAKO, Tokyo, Japan) was used. After two washes, 2 μL of anti-human IgG (FITC-F (ab′) 2 anti-human IgG, DAKO) was cross-reacted in a dark room for 30 min. After washing three times, 500 μL of PBS was added to make a cell suspension, and the fluorescence response patterns were compared using FACS Canto or Fortessa.

### Luminex method

MICA antibody screening was performed using LABScreen MICA Single Antigen-Group 1 (One Lambda Inc.), LABScreen Mixed (One Lambda Inc.) and analyzed using a LABScan 100 flow analyzer (Luminex 100 System) according to the manufacturer’s instructions. The cut-off value of LABScreen Mixed was set to a ratio > 2.7 provided by the manufacturer, and this ratio was calculated by normalizing the bead result value to a negative control value. It was determined through this, and among the total 64 samples, 44 MICA antibodies were determined to be positive and 20 were determined to be negative. The same 64 samples were tested using LABScreen Single Antigen-Group 1 (single antigen) and the mean value + 4 × standard deviation (mean + 4SD) of 20 negative samples was set as the cut-off value. Afterwards, LABScreen MICA Single Antigen-Group 1 was described by the Luminex method.

### Statistical analysis

Statistical analyses were performed using GraphPad Prism 7 (GraphPad, San Diego, CA, USA) software. We analyzed the correlation between the FCM and Luminex methods using Pearson’s correlation analysis. The results were obtained from a single experiment on 64 donors. *P* value ≤ 0.05 was considered significant. The figures were generated using GraphPad Prism 7 and FlowJo v10 (FlowJo LLC, Ashland, OR, USA).
